# Associations Among Diet, Health, Lifestyle, and Gut Microbiota Composition in the General French Population: Protocol for the Le French Gut – Le Microbiote Français Study

**DOI:** 10.2196/64894

**Published:** 2025-05-13

**Authors:** Chloe Connan, Sébastien Fromentin, Mourad Benallaoua, Anne-Sophie Alvarez, Nicolas Pons, Benoît Quinquis, Christian Morabito, Julie-Anne Nazare, Elise Borezée-Durant, Le French Gut Consortium, Florence Haimet, Stanislav Dusko Ehrlich, Karine Valeille, Alexandre Cavezza, Hervé Blottière, Patrick Veiga, Mathieu Almeida, Joël Doré, Robert Benamouzig

**Affiliations:** 1 Université Paris-Saclay, INRAE, MetaGenoPolis (MGP) Jouy-en-Josas France; 2 Department of Gastroenterology Avicenne Hospital, Assistance Publique-Hôpitaux de Paris Université de Paris Bobigny France; 3 Univ-Lyon, CarMeN Laboratory, Inserm, Inrae, Université Claude Bernard Lyon-1 Lyon France; 4 Université Paris-Saclay, INRAE, AgroParisTech, Micalis Institute Jouy-en-Josas France; 5 see Authors' Contributions; 6 INRAE Mica division Jouy-en-Josas France; 7 Nantes Université, INRAE, UMR 1280, PhAN Nantes France

**Keywords:** gut microbiome, health, chronic diseases, symbiosis, gut, French citizen, French, fecal, fecal samples, nutritional, clinical data, Gut microbiota, gut flora, microorganisms, microbiome, relationship, dietary habit, ecosystem, ecosystem tools, innovation

## Abstract

**Background:**

Over the past 2 decades, the gut microbiota has emerged as a key player in human health, being involved in many different clinical contexts. Yet, many aspects of the relationship with its host are poorly documented. One obstacle is the substantial variability in wet-laboratory procedures and data processing implemented during gut microbiota studies, which poses a challenge of comparability and potential meta-analysis.

**Objective:**

The study protocol described here aimed to better understand the relationship between health, dietary habits, and the observed heterogeneity of gut microbiota composition in the general population. “Le French Gut – Le microbiote français” aimed to collect, sequence, and analyze 100,000 fecal samples from French residents using a high-quality shotgun metagenomic pipeline, complemented with comprehensive health, lifestyle, and dietary metadata.

**Methods:**

“Le French Gut – Le microbiote français” is a prospective, noninterventional French national study involving individuals, the creation of a biological collection (feces), and the exploitation of data from questionnaires and the National Health Data System (Système National des Données de Santé). This national study is open to all metropolitan French adult residents, excluding those who have undergone a colectomy or digestive stoma, or who have had a colonoscopy or taken antibiotics in the last 3 months. This is a home-based trial in which volunteers complete a questionnaire with insights about their health and habits, and in which stool samples are self-collected. Data analysis is structured into 6 work packages, each focusing on a specific aspect of the gut microbiome, including its composition and associations with lifestyle, quality of life, and health.

**Results:**

This paper outlines the study protocol, with recruitment having started in September 2022 and expected to continue until the end of December 2025. As of January 2025, a total of 20,000 participants have been enrolled. The first scientific publications based on the data analysis are expected by mid-2025.

**Conclusions:**

“Le French Gut” aims to provide a reference database and new ecosystem tools for understanding the relationship between the gut microbiota, its host, and diet. We expect to be able to find new signatures or targets and promote the design of innovative preventive strategies, personalized nutrition, and precision medicine.

**Trial Registration:**

ClinicalTrials.gov NCT05758961; https://clinicaltrials.gov/study/NCT05758961

**International Registered Report Identifier (IRRID):**

DERR1-10.2196/64894

## Introduction

### Background

The gut microbiota is composed of all the micro-organisms (archaea, bacteria, microeukaryotes, viruses, etc) that inhabit the intestine. It can be considered as an organ in its own that has coevolved with its host in a symbiotic relationship, contributing to the physiological homeostasis known as “symbiosis” [1]. The gut microbiota is a highly complex ecosystem that can be studied at different levels of resolution. This includes examining diversity parameters, characterizing enterotypes [2,3], exploring various taxonomic ranks ranging from the superkingdom to the species level, and even delving into bacterial strain–specific differences. In addition, the functional potential of the microbiota can be assessed, focusing on the metabolic and biochemical capabilities of the gut microbiome and how these are exchanged with the host.

The establishment and evolution of the intestinal microbiota throughout life is multifactorial, with the environment and dietary habits playing a major role [4-10]. Advances in research demonstrate how crucial this symbiosis is for an individual’s health and well-being [11]. Conversely, an imbalance or “dysbiosis” of the gut microbiota is associated with the risk of onset of various chronic diseases, such as those observed in intestinal and liver diseases (inflammatory bowel disease, irritable bowel syndrome, colorectal cancer, nonalcoholic fatty liver disease, and cirrhosis) [6,12,13], obesity [3,14,15], diabetes [16], autoimmune diseases [17-19] and more recently, in neurological diseases via the gut-brain axis [20-26]. Microbial alterations associated with the emergence of pathological conditions affect all microbial strata. Observed alterations include a reduction in microbial diversity [6,13,14,18,19,27], an increase in the proportion of Enterotype B2 [3], as well as disruptions across the different taxonomic ranks [6,13,16,18,19,21,23,25-27]. These alterations extend to their associated metabolic outputs, notably including a reduction in the production of short-chain fatty acids or butyrate [13,15,19,25-27].

Currently, most available microbial studies, including clinical and nutritional data in France and worldwide, are based on relatively small sample sizes, usually ranging from a few dozen to a few hundred individuals, and use different sampling and analysis methods, which makes meta-analysis complex.

To gain a better understanding of the interactions between gut microbiota, diet, and health, it is essential to significantly increase the number of samples of gut microbiota processed in a homogenous manner and cover the widest possible spectrum of phenotypes. For this reason, several large-scale initiatives have emerged, such as “The American Gut project,” launched in 2012, which involves more than 10,000 participants, and more recently the Million Microbiomes from Humans Project (MMHP), launched in 2019, which involves several countries around the world and aims to sequence 1 million metagenomes. Building on this momentum, “Le French Gut” was launched in September 2022 with the aim of describing the gut microbiota and associated nutritional and clinical data of 100,000 major individuals living in France, and the ambition to accelerate microbiota science, a source of innovation for tomorrow’s medicine and nutrition.

### Aims of the Study

By collecting 100,000 gut microbiota profiles combined with metadata on diet, health, and lifestyle, “Le French Gut – Le Microbiote Français” aims at (1) better understanding the heterogeneity of healthy gut microbiota and its link with nutrition, lifestyle, and anthropometric characteristics and (2) predicting changes in gut microbiota associated with diseases such as chronic diseases, neurodevelopmental disorders, and neurodegenerative conditions.

## Methods

### Study Design

“Le French Gut – Le Microbiote Français” is a prospective, noninterventional national study collecting human fecal samples and connecting them with data from self-filling questionnaires and from the Système National des Données de Santé (SNDS). The trial was conducted according to the recommendations of Standard Protocol Items for Clinical Trials 2013 [28]. The project aims at collecting and analyzing 100,000 stool samples from adult volunteers living in France. The duration of this inclusion phase is estimated at 5 years, and the study is planned to run for 20 years, with active (additional questionnaires) and passive follow-up thanks to its link with the SNDS. Ancillary studies could be linked to this main project during this period, including extending it to French overseas departments and territories and to minors.

**Figure 1:**
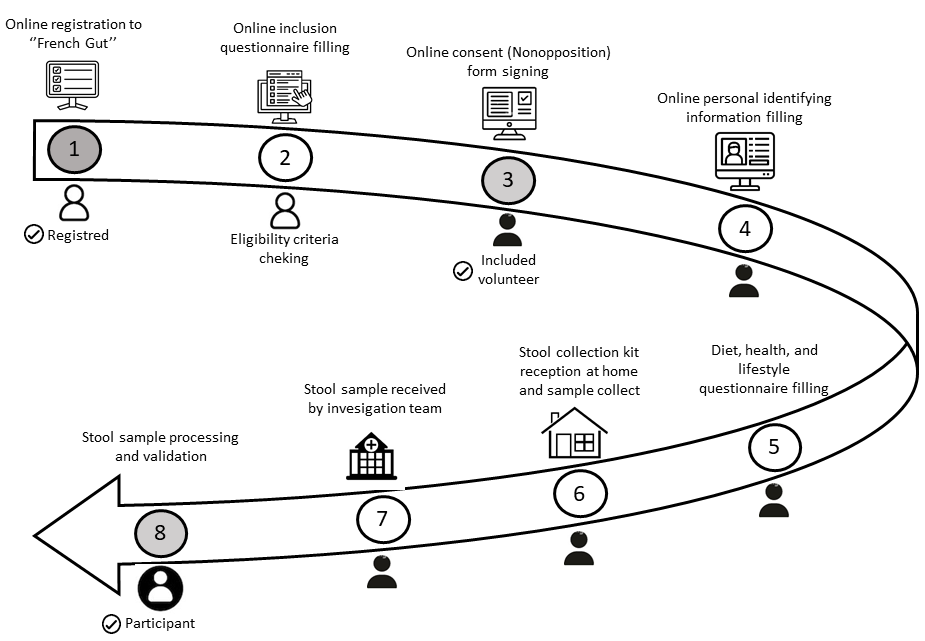
Le French Gut’s volunteer timeline. The timeline starts with the profile registration. After filing the noninclusion form, the nonopposition form, the personal identifying form, the registered becomes a volunteer. The volunteer becomes a participant once the questionnaire has been completed and the stool sample returned to the investigation team.

The study is being carried out in the general population targeting adult volunteers. Participation in the project is free of charge and takes place exclusively via the Le French Gut website [29]. Volunteers are informed about the study through various communication campaigns (national and local media, as well as social networks; Multimedia Appendix 1) and are invited to register on the dedicated website. Questionnaires and forms can be filled in using a user-friendly web interface where all data and personal information security measures are guaranteed, in particular by hosting data in a secure sovereign Health Data Hosting (HDS) certified cloud and General Data Protection Regulation compliant. After reading the information note, volunteers are asked to sign an electronic informed consent form. This procedure generates a permanent inclusion identifier. To become a participant, volunteers must follow the participant’s journey (Figure 1). After verifying eligibility for the study, the participant is asked to complete a 53-item questionnaire about their health, lifestyle, and dietary habits (generally completed in 15 minutes as mentioned in the information note). Once the questionnaire has been completed, a stool sampling kit is automatically sent to the volunteer’s address. The volunteer collects the stool sample at home using the kit and then sends the stool sample tube to the L’Assistance Publique - Hôpitaux de Paris laboratory via regular mail using a prestamped envelope approved for biological material shipment. Once the integrity and quality of the sample have been checked, the samples are pseudonymized and sent to an INRAE (French National Research Institute for Agriculture, Food, and the Environment) laboratory for biobanking and metagenomic analysis, under a secured ISO (International Organization for Standardization) 9001 and ISO 27001 certified pipeline.

It will also be possible to supplement the basic protocol with various questionnaires within ancillary protocols, targeting either the whole population or selected subsamples according to a particular phenotype (age, gender, area of residence, health status, etc).

Participants will have access to the project’s collective scientific feedback, on the website or their personal web page, and will be kept informed of project progress via regular newsletters.

### Primary Outcome

The primary outcome is to characterize the heterogeneity and diversity of the gut microbiota of individuals living in France.

The gut microbiota of participants in the “French Gut” project will be characterized by describing the presence and abundance in the samples of each gut microbial genes listed in our up-to-date gene catalog [30]. From these data, the presence and abundance of the different taxonomic ranks will be inferred, ranging from a fine granularity at species and strain level to a more “zoomed out” phylum level. The richness and diversity of the microbiome (genes and species) will be described. The functional modules, corresponding to the metabolic pathways encoded by the species detected in each sample, will be determined.

### Secondary Outcomes

The secondary outcomes are to study the variations in the gut microbiota of participants as a function of microbial profile, age, sociodemographic and anthropometric characteristics, lifestyle and eating habits, presence of known pathologies at inclusion, occurrence during follow-up of certain pathologies identified by analysis of data from health questionnaires and SNDS data.

### Recruitment

The recruitment of participants is mainly based on a direct approach with “public” communication around the project. Participants learn about the project “Le French Gut” through various communication campaigns, including social media, television, radio, national and regional newspapers, magazines, and events. Volunteers can find all the information they need to take part in the project on the website [29]. Communication also aims to raise awareness on the intestinal microbiota, its link with health, and how to care for it through diet. To this end, several communication tools and podcasts are broadcast to the general public and widely distributed on social media.

Partnerships with existing cohorts will also enable their members to take part in this project. Specific populations, such as members of associations or networks, may be included once the scientific committee has validated the interest of these targeted recruitments. Registration procedures for individuals recruited in this way will be similar to those for the general public.

### Eligibility Criteria

Le French Gut is a national contribution open to anyone meeting the eligibility criteria. Eligible individuals are adult men and women living in mainland France, with an electronically signed participation agreement. Detailed inclusion and noninclusion criteria are presented in Textbox 1. These eligibility criteria may be different in ancillary projects.

**Le French Gut eligibility criteria.**

**Inclusion criteria**

- Adult men and women residing in mainland France
- Electronically signed consent form

**Noninclusion criteria**

- Nonadult person
- Person not living in France
- Persons subject to a protective measure, in particular, under guardianship or curatorship or unable to express their consent
- Person underwent a colectomy
- Person with a digestive stoma
- Person who has not signed the consent
- Person who did not answer to the inclusion questionnaire
- Person who did not send a compliant stool sample
- Person having taken antibiotic in the 3 months before inclusion
- Person who underwent a colonoscopy in the 3 months before inclusion

### Sample Size

The sample size was calculated taking into account the main objective, which is to specify the presence and the abundance of over 2500 microbial species of the digestive tract and, in particular, the number of subjects required to characterize the enterotype B2 [3]. The enterotype B2 is a fairly rare ecological configuration of the microbiota that is most frequently associated with low-grade inflammation, higher prevalence of cardiometabolic conditions, and predictive of nonresponse to a diet designed to promote weight loss. According to a power test performed on French fecal samples from the MetaCardis cohort, with a population of 100,000 individuals and an expected prevalence of enterotype B2 of 5%, we will have the statistical power needed to characterize 74% of the microbiome that makes up the enterotype B2 with an α risk of 0.05 and β risk 0.80. A total of 100,000 participants will provide sufficient statistical power to assess the link between the composition of the gut microbiota and diseases of interest, but also to assess the composition of the intestinal microbiota of asymptomatic individuals at high risk of developing a disease. Indeed, diseases of interest include type 2 diabetes (prevalence of 6.4% in 2022), heart disease (8.3%), long-term psychiatric disease (4.1%) but cancer (2.3%) [31].

### Ethical Considerations

#### Human Subject Ethics Review Approvals or Exemptions

This study has been approved by the the “Comité de Protection des Personnes” Sud-Est IV (CPP, 21.00225.000006) and the “Commission Nationale Informatique et Liberté” (CNIL, DR-2022-141).The study registered in France under reference 2021-A01439-32 was authorised on October 15,2021. Detailed trial registration dataset is presented in Multimedia Appendix 2.

#### Informed Consent

All participants were asked for their consent to participate in this study before enrollment and agree to its privacy policy and terms of service required for the remote collection of their information. The participation is entirely voluntary. Electronic informed consent form to participate is obtained from all study participants. Participants can opt out of the study anytime by going on its account. The participant may withdraw from the study at any time without justification (under the “management of my participation” section in his personal space). The participant is aware that the withdrawal will not affect any activities conducted or the use of data obtained based on the informed consent given before the withdrawal, in accordance with the Public Health Code. Furthermore, the participant consents to the reuse of his data under pseudonymized in the case of a study extension, or for ancillary studies within the same research field (microbiota), and only after approval of relevant authorities.

Participants have access to all studies in which their data have been used, and can withdraw their participation at any time from any study.

#### Privacy and Confidentiality

After logging into the French Gut registration site, participants will be assigned a pseudonym that will be created using the anonymization or pseudonymization techniques recommended by the National Agency for the Security of Information Systems and the French Data Protection Authority (CNIL). This pseudonym is guaranteed to be nonidentifiable. The reference is unique and will be retained for the entire duration of the study. Furthermore, the results will be reported only in aggregate form and not on an individual basis.

#### Compensation Details

Participants will be kept informed about the progress of the study and its overall results through regular newsletters, shared on participants’ accounts, as well as through webinars exclusively dedicated for them. Participation in this research is voluntary and does not offer any possibility of personal benefit, nor does it entitle participants to any compensation, except the one mentioned above.

### Participant Journey

To take part, volunteers need to answer a 53-item online questionnaire about their health, lifestyle, and dietary habits, and collect a compliant stool sample. The participant’s journey is detailed in Figure 1.

#### Registration and Questionnaire

To take part in the study, volunteers need to create a personal account on the project’s website [29]. Registration and data collection take place directly on the project website, using an end-to-end encrypted connection. The questionnaire and data are stored in an HDS-certified environment (SKEZIA), provided by a company specializing in eHealth solutions and e-cohorts. The entire platform was initially developed and tested in a development environment before being deployed in production and made accessible to participants. After checking that the volunteer is eligible and has signed the informed consent form, the volunteer completes the questionnaire, which consists of 53 items divided into 3 different parts (Multimedia Appendix 3). The first part of the questionnaire provides sociodemographic data (duration of residence in mainland France, mode of delivery, etc) as well as lifestyle data (smoking habits, sedentary lifestyle, and physical activity). The second part of the questionnaire provides information on anthropometric characteristics (weight and height) used to calculate the BMI. This section also collects health data, including chronic diseases. In order to characterize the digestive health of the volunteers, the questionnaire also includes questions relating to gastrointestinal symptoms and factors influencing digestive health. This questionnaire also includes the Bristol Stool Consistency Scale [32]. It also contains questions to define an assessment of a participant’s mental well-being called the “Perceived Stress Scale 4” or PSS-4 [33]. The third part of the questionnaire is devoted to the dietary habits. First, food exclusions and specific types of diet are addressed. The volunteers then indicate, for each of the food families present in the questionnaire, the frequency of their usual daily or weekly consumption. In addition, the questionnaire focuses on the specific dietary factors that have an impact on the gut microbiota. Overall, the 53 questions are distributed across 4 web pages. These questionnaires are adaptive, and each page contains an average of 16 questions (ie, the request for clarification on certain items only appears if the participant answered “yes” to the first question, for example, to provide a specific digestive disease). Full completion of the questionnaire is mandatory to move on to the next stage, that is, receiving the kit at home. Volunteers can modify their answers as long as they have not submitted the questionnaire. Once submitted, the participant can contact the investigation team at any time to indicate whether any of the eligibility criteria have changed (ie, antibiotics taken between completion of the questionnaire and sample collection), or to modify their responses after the submission of the questionnaire. The questionnaire is a one-shot and can only be completed once.

As the project progresses, additional questionnaires will be offered to participants who have agreed to be recontacted for further contribution to scientific explorations. These questionnaires will be more detailed on health, lifestyle, and dietary habits. They will not be mandatory, but they will help us to answer specific scientific questions that emerge as the project progresses.

The validity of the data is verified by input and consistency checks. The questionnaire database is stored in an HDS environment and the metagenomic data on the MGP’s information technology infrastructure, as close as possible to the sequencing instruments and the heavy bioinformatics processing.

#### Stool Sampling

After completing the initial questionnaire, a stool sampling kit is sent to the volunteer. The stool sample is collected at home by the volunteer in a tube containing a stabilizing liquid buffer (DNA and RNA shield). The date and time on which the sample was taken is handwritten by the volunteer on the pouch containing the tube. After sampling, the tube is mailed directly to the investigation team via a prepaid envelope. The investigation team checks the conformity of the sample (eg, presence of identification data on tube, missing sample, and broken tube). Samples that do not pass the initial quality control are flagged up and a new sampling is scheduled. Quality control–validated samples are stored at –80 °C at the MGP-SAMBO BRC (Biological Resources Center), which is ISO 9001–certified and labelled GIS-IBiSA (CRB N°242), until further processing.

### Stool Sample Processing

#### DNA Extraction and Shotgun Sequencing

Total fecal DNA will be extracted using an in-house automated protocol that has been developed and improved on the International Human Microbiome Standards (IHMS) standard procedures (SOP 07 [34]). Extracted DNA is evaluated by fluorometric quantification, purity assessment, and fragment size analysis, then if suitable, sequenced using a high-throughput short-read DNA sequencer. A minimum of 2×20 million paired-end reads of 150 nucleotides is produced for each sample.

#### Microbial Gene Abundance Table and Taxonomical Profiling

Sequencing data are cleaned to (1) remove remaining sequencing adapters, (2) trim low-quality reads, and (3) discard reads too short (<60 bp) using Alien Trimmer (v0.4.0) or equivalent quality trimming software [35]. Reads mapping to the human genome are removed using Bowtie2 (v2.5.4) [36]. Microbial gene abundance and taxonomical profiling are then performed using Meteor2 [37] with our up-to-date gene microbial genes [30] and species (clustered with MSPminer [38]) catalogs of the human microbiota. Gene abundance profiling table is generated by mapping the reads onto the genes catalog. Species abundances are estimated as the mean abundance of the species marker genes. Richness and diversity parameters are computed using the species abundance table with Vegan R package [39]. Our catalogs will be continuously updated throughout the project based on newly identified clustered species, newly available taxonomy, gene annotations, and genomes.

### Contribution of the SNDS

The French national health data system also referred to as SNDS contains individual data for all reimbursements of health expenses for more than 99% of residents of French territory, that is, more than 65 million people. This system enables powerful analyses both in terms of the number of subjects involved and the hindsight now available (data collected since 2007). Available data are both administrative and medical. Administrative data are mainly sociodemographic data (age, sex, complementary universal health cover [named CMU-c], etc). Medical data include, among others, the list of chronic diseases of the volunteers that are 100% reimbursed by the French medical system or, for example, the exhaustive list of reimbursed drugs that have been prescribed over the years. Collecting the social security number during inclusion allows direct matching between the SNDS data and gut microbiota data from Le French Gut study.

This matching will allow us to gain a deep insight on the connection between the present participant’s health and its gut microbiota. It will also bring light on how the participant’s medical history is connected to its present gut microbiota profile.

### Statistical Methods

The statistical plan is designed to address both primary and secondary outcomes. It is structured into 6 distinct work packages, each targeting key aspects of the gut microbiome and its interactions.

First, microbial ecology and variability: this work package aims to characterize microbial ecology, variability, and composition across the French population. The main objective is to consolidate validated ecological tools and develop new ones leveraging the statistical power of this study. These tools will capture microbial variations at multiple levels, including alpha and beta diversity metrics, enterotypes [2], local partitioning [40], microbial guilds, and taxonomic ranks down to the strain level. These ecological tools will be analyzed across the entire French population. In addition, this work package will investigate how regional environmental exposures (air, water, soil, etc) correlate with microbiome variations.

Second, gut microbiome in healthy individuals: this work package focuses on establishing baseline profiles of the gut microbiome in healthy individuals. The first objective is to define and characterize a healthy population based on lifestyle habits, dietary patterns, and quality of life. Clustering techniques will be applied to classify individuals based on lifestyle factors (eg, smoking, alcohol consumption, and physical activity), dietary habits, and quality-of-life indicators (eg, sleep quality, digestive symptoms, and perceived stress). These clusters will allow us to explore whether the microbiome can reflect and influence the accumulation of risk factors in the healthy French population. The second objective is to assess the predictive potential of the microbiome for future health outcomes. Using the SNDS database, we will evaluate whether participants develop pathologies over a 10-year follow-up and identify microbiome signatures associated with disease onset.

Third, this work package investigates the relationship between the gut microbiome and the presence of pathological conditions. The pathological population will be characterized in terms of lifestyle habits, dietary patterns, and existing conditions. The project’s extensive statistical power will enable the stratification of pathologies, facilitating validation of known microbial signatures and identification of novel microbial markers linked to disease progression.

Fourth, this work package explores interactions between dietary patterns and microbiome composition. The first step involves characterizing dietary habits within the French population, both globally and with a focus on specific food components such as proteins, fibers, and polyphenols. We will then analyze how these dietary patterns influence and are influenced by the gut microbiome.

Fifth, this work package focuses on characterizing nonbacterial components and subdominant members of the microbiome. The large sample size will enable the exploration of less-studied gut ecological niches and microbes, including archaea, viruses, fungi, and protozoa. Their distribution, abundance, and associations with lifestyle, quality of life, and health conditions will be examined. In addition, we will investigate subdominant microbial species and strains that remain unexplored due to limited sample sizes in previous studies.

Sixth, this work package examines the influence of environmental exposures, including antibiotic and other medication use, on the microbiome. The first step involves stratifying the population based on medication and antibiotic usage. We will then assess microbiome responses to these exposures, with a specific focus on antibiotics. Thanks to the SNDS database, we will analyze antibiotic exposure data spanning the 20 years before participants’ enrollment (ie, up to 2006), allowing an in-depth investigation of the relationship between multiple antibiotic exposures and antibiotic resistance in the gut microbiome.

Given the explanatory nature of Le French Gut, each work package statistical plan will be further refined as data exploration progresses.

Each work package will use comparable statistical tools, methods, and analytical approaches. Except for work packages 1 and 5, the initial statistical step in each work package consists of characterizing the data collected from all included volunteers. Descriptive statistics (eg, mean and SD for continuous variables and contingency tables for categorical variables) will be used to summarize the data. Graphical visualizations, univariate analyses (eg, *t* tests and ANOVA), and multivariate analyses (eg, principal component analysis, factorial analysis for mixed data, and network analysis) will be performed to explore data structure, detect outliers, and identify potential stratifications.

For univariate analyses, conventional statistical tests (eg, Mann-Whitney-Wilcoxon, *t* test, Kruskal-Wallis, and chi-square tests) will be used to compare microbial, dietary, sociodemographic, and clinical variables across predefined volunteer groups. *P* values will be adjusted using the Benjamini-Hochberg procedure to control the false discovery rate. Correlation analyses will be conducted to examine associations between microbial species of interest and sociodemographic, lifestyle, environmental, nutritional, and clinical parameters.

Multivariate analyses will serve both descriptive and predictive purposes. Descriptive multivariate methods such as principal component analysis, factorial correspondence analysis, clustering, multidimensional scaling, and bacterial co-occurrence network analyses will be used to structure and synthesize the data. Specific complexity-reduction approaches include enterotype classification, which discretizes microbial heterogeneity into a limited number of states [2], and microbial guilds, which identify clusters of species with coabundance patterns across conditions. In addition, unsupervised clustering methods will be used to define microbiome-based subpopulations independently of phenotypic characteristics, allowing unbiased subgroup discovery.

For predictive modeling, supervised learning techniques such as discriminant analysis, random forests, and regression models will be applied to assess the microbiome’s ability to predict phenotypes of interest, including disease presence, nutritional profiles, environmental exposures, and health trajectories. These models will be trained and validated using independent test subsets to evaluate their predictive performance.

### Integration of the “Le French Gut” Project Into the MMHP

Metagenomic data from the “Le French Gut” will be shared anonymously as part of the collaborative science project (MMHP) which involves many countries worldwide including France, Sweden, Denmark, Latvia, and China as founding members. This project aims at sequencing one million human microbial metagenomes coming from the gut, mouth, skin, and other human organs. It will make available to researchers the metagenomic data associated with the sampling date, the sampling site, and protocols as well as a limited amount of metadata, such as age, sex, BMI, geographical location (France for this study) and “healthy” or “ill” status (with *ICD-10* [*International Statistical Classification of Diseases, Tenth Revision*] disease code if available).

## Results

Recruitment started in September 2022. As of January 2025, we enrolled 20,000 participants. The first scientific publications based on the data analysis are expected by mid-2025.

## Discussion

### Anticipated Findings

Le French Gut is a national participatory science project designed to explore the heterogeneity of gut profiles within the French resident population with an ambitious statistical power, 100,000 subjects. By leveraging robust statistical methods and state-of-the-art bioinformatics tools, Le French Gut seeks to offer novel insights into the definition and organization of the gut microbiome. While the project intends to shed light on its variations within the national population, it also aims to provide a better understanding of the shifts potentially associated with the onset of pathological conditions. However, the full implications of these shifts and their direct link to specific health outcomes will require further investigation.

One of the main advantages of this protocol is the simplicity of the participant route. Everything can be done at home. This ease of use, coupled with large public communication and awareness campaigns, is an asset toward reaching 100,000 participants. Like the MMHP, The American Gut, The British Gut, The LifeLines Deep, The 10K, or also The Flemish Gut projects, Le French Gut is part of this worldwide willingness to drastically increase the available number of gut microbiota profiles. Le French Gut will constitute a significant database with 100,000 shotgun-sequenced gut microbiota profiled up to the strain levels. In addition to the microbiome data, information on participants’ lifestyles, medical history, and a detailed characterization of their dietary habits will also be included. While this comprehensive dataset has the potential to provide valuable insights, the complexity of interpreting such data means that drawing definitive conclusions about the relationships between microbiota, lifestyle, and health outcomes will require careful analysis and further research. Another main strength of Le French Gut is the high-quality standards used to biobank and process all microbial samples. One of the main biases when comparing metagenomics projects is the absence of sample processing information and quality measures between the projects being compared, whether in terms of sampling, storage, extraction, or sequencing. Within the Le French Gut project, the 100,000 microbiome profiles will be processed with protocols that will follow the IHMS [41] or higher standards that will arise during the course of the project. In addition, Le French Gut’s flexible recruitment process allows an interesting opportunity for synergistic collaborations with other cohorts aimed at deeply characterizing patients or healthy subjects. This strategic approach will facilitate the gathering of valuable data and promote cross-cohort research initiatives. In addition to the core project, we plan to extend the recruitments to children and teenagers (Le French Gut Kids) and to overseas territories.

Le French Gut will contribute to a better understanding of the organization of the intestinal microbiota and the factors related to its variations. Thanks to its statistical power, Le French Gut will have to potential to help validate well-known ecosystemic tools (diversity measures, enterotypes, etc) and confirm well-known discoveries. It will also provide an opportunity to deploy novel tools, such as microbial guilds, gut microbiota global, and local partitioning [40] at a larger scale, and new microbial metabolic reconstruction pathways. In conjunction with nutritional data, these ecosystemic tools offer an opportunity to explore the connection between diet and the diverse range of ecological states of the human gut microbiota. With this project, we anticipate to lay the foundation for precision nutrition approaches aimed at defining diets or supplements tailored to specific gut microbiota states. In addition, Le French Gut will provide the scientific community with valuable insights into the heterogeneity of microbial profiles within a healthy population, offering a clearer understanding of how the gut microbiota may potentially serve as a predictor for future health issues. Thanks to the SNDS, gut microbiota profiles will be integrated into each participant’s overall past, present, and future health landscape. We expect to discover new biomarkers that could lead to possible new preventive strategies through personalized nutrition, diagnostics, or therapeutics. Finally, Le French Gut is expected to provide recommendations for more systematic use of the microbiota as a tool to support clinical management.

### Conclusions

“Le French Gut” aims at providing a reference French gut microbiome database and new ecosystem tools for understanding the relationship between the gut microbiota, its host, and diet. We expect to be able to find new signatures or targets and promote the design of innovative preventive strategies, personalized nutrition, and precision medicine.

## Appendix

Multimedia Appendix 1 Flyer Le French Gut.

Multimedia Appendix 2 Trial status.

Multimedia Appendix 3 Le French Gut main questionnaire.

Multimedia Appendix 4 Executive and steering committee.

## Abbreviations

CNIL: Comité National Informatique et Liberté
HDS: Health Data Hosting
ICD-10: International Statistical Classification of Diseases, Tenth Revision
IHMS: International Human Microbiome Standards
INRAE: French National Research Institute for Agriculture, Food, and the Environment
ISO: International Organization for Standardization
MMHP: Million Microbiomes from Humans Project
SNDS: Système National des Données de Santé

## Data Availability

The datasets analyzed during this study and statistical code are available from the corresponding author on reasonable request, as is the whole protocol.
