# Correction: Associations Among Diet, Health, Lifestyle, and Gut Microbiota Composition in the General French Population: Protocol for the Le French Gut – Le Microbiote Français Study

**DOI:** 10.2196/80127

**Published:** 2025-07-14

**Authors:** Chloe Connan, Sébastien Fromentin, Mourad Benallaoua, Anne-Sophie Alvarez, Nicolas Pons, Benoît Quinquis, Christian Morabito, Julie-Anne Nazare, Elise Borezée-Durant, Le French Gut Consortium, Florence Haimet, Stanislav Dusko Ehrlich, Karine Valeille, Alexandre Cavezza, Hervé Blottière, Patrick Veiga, Mathieu Almeida, Joël Doré, Robert Benamouzig

**Affiliations:** 1 Université Paris-Saclay, INRAE, MetaGenoPolis (MGP) Jouy-en-Josas France; 2 Department of Gastroenterology Avicenne Hospital, Assistance Publique-Hôpitaux de Paris Université de Paris Bobigny France; 3 Univ-Lyon, CarMeN Laboratory, Inserm, Inrae, Université Claude Bernard Lyon-1 Lyon France; 4 Université Paris-Saclay, INRAE, AgroParisTech, Micalis Institute Jouy-en-Josas France; 5 see Authors' Contributions; 6 INRAE Mica division Jouy-en-Josas France; 7 Nantes Université, INRAE, UMR 1280, PhAN Nantes France

In “Associations Among Diet, Health, Lifestyle, and Gut Microbiota Composition in the General French Population: Protocol for the Le French Gut – Le Microbiote Français Study” ([JMIR Res Protoc 2025;14:e64894]) several errors appeared in the published version related to the group authorship of Le French Gut.

1. The affiliations of contributors from public institutes and private companies were omitted in the published version and have been included in the corrected version as:

Monique Axelos (INRAE), Sylvie Binda (Lallemand Health Solutions), Pierre Cressard (Nahibu), Anne-Marie Davila-Gay (Université Paris-Saclay, AgroParisTech, INRAE, UMR PNCA), Christophe d’Enfert (Institut Pasteur, INRAE), Assia Dreux-Zigha (Greentech), Erik Eckhardt (DSM-Firmenich), Etienne Formstecher (GMT Science), Maiana Houssaye (INSERM), Milan Lazarevic (APHP), Sophie Legrain (Lesaffre), Oana Bernard (Biocodex), Benoît Fouchaq (Biofortis), Pedro H. Oliveira (Génomique Métabolique, Genoscope, Institut François Jacob, CEA, CNRS, Université d’Évry, Université Paris-Saclay), Raish Oozeer (Danone Nutricia Research), and Karine Roget (NexBiome Therapeutics).

2. The list of names under the Le French Gut Consortium in the “Authors' Contributions” section were revised as follows:

Françoise Levacon and Marie-Emmanuelle Le Guern

were replaced with

Benoît Fouchaq and Oana Bernard, respectively

3. The location “Gif-sur-Yvette” was incorrectly listed in the affiliation for the Le French Gut consortium and has been removed in the corrected version.

The correction will appear in the online version of the paper on the JMIR Publications website, together with the publication of this correction notice. Because this was made after submission to PubMed, PubMed Central, and other full-text repositories, the corrected article has also been resubmitted to those repositories.

