# Supplementary material for: Associations Among Diet, Health, Lifestyle, and Gut Microbiota Composition in the General French Population: Protocol for the Le French Gut – Le Microbiote Français Study
Source: JMIR Res Protoc. 2025 May 13;14:e64894. doi: 10.2196/64894 (PMC12117270; doi:10.2196/64894)
Supplement: Multimedia Appendix 1 [file resprot_v14i1e64894_app1.pdf]

Le microbiote français

Le French Gut

Ensemble,
faisons avancer la science du microbiote intestinal :
participez au projet Le French Gut !

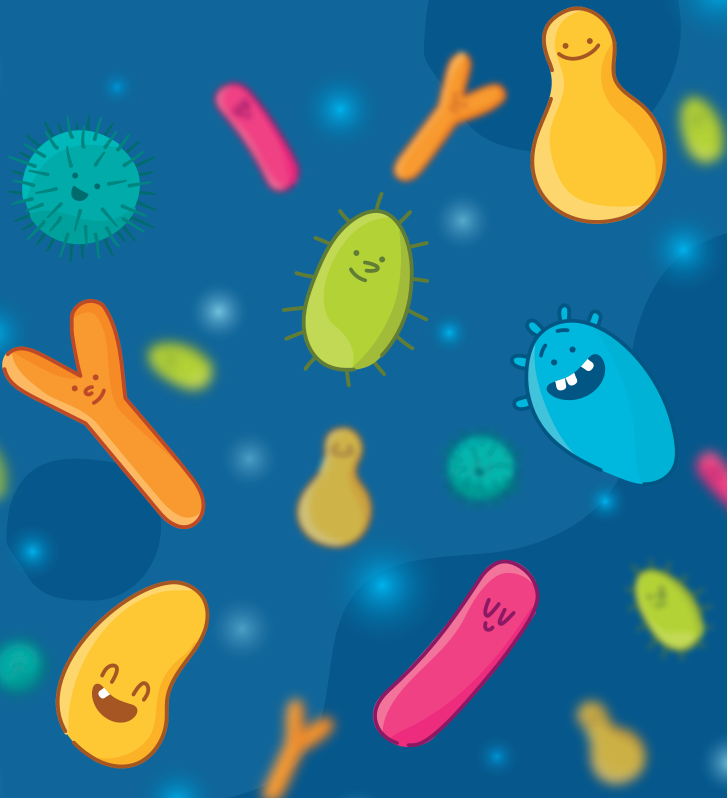

UN PROJET INRAE | metagenopolis mgps.eu | FRANCE 2030 | ASSISTANCE PUBLIQUE HÔPITAUX DE PARIS

Le French Gut

Contribution nationale visant à **collecter et analyser les selles de 100 000 participants** ainsi que les données nutritionnelles et cliniques associées d'ici 2027 pour **mieux comprendre ce que sont des microbiotes intestinaux sains et leurs modifications en cas de maladies.** Le French Gut, porté par INRAE, est mené en partenariat avec des institutions publiques et acteurs privés, impliqués dans le domaine du microbiote.

Pourquoi participer ?

Avec votre aide, nous pourrons réaliser de grandes avancées dans la recherche sur le microbiote intestinal pour **ouvrir la voie à des thérapies innovantes dans le contexte des maladies chroniques** (diabète, obésité, cancer...). **Vous n'aurez pas de retour individuel** sur votre microbiote mais aurez **accès aux résultats collectifs du projet et l'opportunité d'interagir avec nos experts** lors de webinaires dédiés.

Comment participer ?

En remplissant un questionnaire et en faisant un don de selles. **La participation se fait exclusivement par le site internet Le French Gut** dans un environnement sécurisé. Elle est **ouverte à toute personne majeure et volontaire, résidant en France métropolitaine**, sous réserve d'éligibilité. Faites la différence pour améliorer la qualité de vie du plus grand nombre, **devenez un participant Le French Gut !**

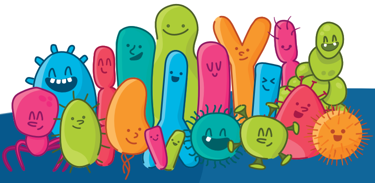

Ne pas jeter sur la voie publique

Le microbiote français
Le French Gut

Imprimé par Easyflyer (www.easyflyer.fr) sur du papier issu de fibres recyclées

MetaGenoPolis est une unité experte dans la recherche du microbiote intestinal de INRAE, établissement public à caractère scientifique et technologique
