# Supplementary material for: Associations Among Diet, Health, Lifestyle, and Gut Microbiota Composition in the General French Population: Protocol for the Le French Gut – Le Microbiote Français Study
Source: JMIR Res Protoc. 2025 May 13;14:e64894. doi: 10.2196/64894 (PMC12117270; doi:10.2196/64894)
Supplement: Multimedia Appendix 2 [file resprot_v14i1e64894_app2.docx]

Appendix 1 - Le French Gut Trial details

| **Data Category** | **Information** |
| --- | --- |
| Official Title | The French Gut : Le microbiote français |
| NCT Number | NCT05758961 |
| Other Study ID Numbers | 2021-A01439-32 |
| Current Responsible Party | Institut National de Recherche pour l'Agriculture, l'Alimentation et l'Environnement |
| Current Study Sponsor | Institut National de Recherche pour l'Agriculture, l'Alimentation et l'Environnement |
| Collaborators | Assistance Publique - Hôpitaux de Paris |
| Investigators | Principal Investigator:  Robert BENAMOUZIG, Pr  AP-HP  Study Chair:  Joël DORE, Pr  INRAE |
| Recruitment Status | Recruiting |
| Enrollment /Target Sample Size | 100000 |
| Study Start Date | 2022-09-15 |
| Primary Completion Date | 2025-12-31 |
| Study Completion Date | 2042-09-15 |
| Eligibility Criteria | Inclusion Criteria:   - Men or women over the age of 18 living in France. - Consent form signed electronically.   Non inclusion Criteria:   - Non-adult person (declarative); - Person not living in France (declarative); - Persons subject to a protective measure, in particular under guardianship or curatorship or unable to express their consent (declarative); - Person having had a colectomy (declarative); - Person with a digestive stoma (declarative); - Person who has not signed the consent; - Person who did not answer the entry questionnaire; - Person who has not sent a compliant stool sample; - Antibiotic intake in the 3 months before inclusion (declarative); - Performing a colonoscopy in the 3 months preceding inclusion (declarative). |
| Location Countries | France |
| Study type | Observational |
| Primary outcome(s) | Heterogeneity and diversity of the gut microbiome of 100,000 subjects residing in France |
| Key secondary outcomes | Variations of the gut microbiome according to age, socio-demographic and anthropometric characteristics, lifestyle and dietary habits or the presence of known diseases at inclusion |
