# Supplementary material for: Associations Among Diet, Health, Lifestyle, and Gut Microbiota Composition in the General French Population: Protocol for the Le French Gut – Le Microbiote Français Study
Source: JMIR Res Protoc. 2025 May 13;14:e64894. doi: 10.2196/64894 (PMC12117270; doi:10.2196/64894)
Supplement: Multimedia Appendix 3 [file resprot_v14i1e64894_app3.docx]

A propos de vous :

1. Quel a été votre mode de naissance ?

* Accouchement par voie basse / par césarienne / Je ne sais pas

2. Êtes-vous né de façon prématuré (c'est-à-dire entre 6 et 8 mois de grossesse) ?

* Oui / Non / Je ne sais pas

3. Quel a été votre mode d'alimentation à la naissance?

*Allaitement maternel -- Alimentation au biberon exclusivement -- Je ne sais pas

4. Quel est le diplôme le plus élevé que vous ayez obtenu ?

* Aucun diplôme ou certificat d’études primaires -- Brevet des collèges -- CAP, BEP ou équivalent -- Baccalauréat, brevet professionnel ou équivalent -- Diplôme du supérieur court (niveau bac + 2) -- Diplôme du supérieur long (supérieur à bac + 2) -- Autre (précisez : _________)

5. Combien de personnes vivent dans votre foyer, y compris vous-même ?

6. Depuis combien d'années (en continu) résidez-vous en France à ce jour (hors voyages de moins d'un mois) ?

* Nombre d'années à indiquer

7. Consommez-vous du tabac ?

* Non-fumeur -- Ancien fumeur – Fumeur actuel : < 5 cigarettes/jour -- 5-10 cigarettes/jour -- 11-20 cigarettes/jour -- > 20 cigarettes/jour -- cigarettes électronique (avec nicotine)

8. Au cours des 4 dernières semaines, quelle est la durée moyenne de votre exposition aux écrans (téléphones portables, tablettes, ordinateurs, télévision, etc.) ?

* Jamais -- Rarement < 1 h/jour -- Occasionnelle 1 à 2 h/jour -- Régulière 2 à 4 h/jour --Quotidienne > 4 h/jour

9. Comment qualifieriez-vous votre temps hebdomadaire d'activité physique modérée ou intense (la marche y compris) ?

* Quotidien / Régulier 3-5 fois/semaine, Occasionnel 1-2 fois par semaine, Rarement quelque fois /mois, Jamais

10. Êtes-vous en contact régulier avec des animaux (possession d’un animal de compagnie, travail quotidien avec des animaux, etc.)?

*Oui -- Non

*Si oui, veuillez préciser le(s)quel(s)

Votre santé :

11. Avez-vous déjà été diagnostiqué positif à la COVID ?

* Non / Oui

*Si Oui : le diagnostic d’infection COVID a été réalisé :

Depuis moins de 3 mois / Entre 3 et 6 mois / Depuis plus de 6 mois

12. A quand remonte votre dernière prise d’antibiotique ?

* Je n’ai pas pris d’antibiotique dans l’année / Comprise entre 6 mois et 1 an / Comprise entre 3 et 6 mois / Inférieure à 3 mois

13. Quel est votre poids actuel en kg ?

* kg à indiquer

14. Quelle est votre taille en cm ?

* cm à indiquer

15. Pour les femmes, Êtes-vous enceinte ?

* Oui / Non / Je ne sais pas

16. Souffrez-vous d'une maladie chronique diagnostiquée par un médecin ?

* Oui / Non / Je ne sais pas.

* Si oui, menu déroulant Menu déroulant :

1- Maladies respiratoires : Asthme, Bronchite chronique (BPCO), Insuffisance respiratoire chronique grave, Tuberculose active en cours de traitement, Mucoviscidose, Emphysème, Autre.

2- Hypertension artérielle et autres maladies cardiovasculaires : Hypertension artérielle, Accident vasculaire cérébral, Artériopathies chroniques avec manifestations ischémiques, Insuffisance cardiaque grave, Cardiopathies valvulaires graves, Troubles du rythme graves, Cardiopathies congénitales graves, Infarctus coronaire, Autres.

3- Maladies métaboliques : Diabète de type 1, Diabète de type 2, Maladies métaboliques héréditaires nécessitant un traitement prolongé spécialisé, Hypothyroidie ou hyperthyroidie, Autres.

4- Pathologies Digestives : Maladies chroniques actives du foie et cirrhose, Stéatose hépatique (NAFL ou NASH), Antécédent d'ulcère duodénal ou gastrique, rectocolite hémorragique et maladie de Crohn évolutives, Polypes coliques, Syndrome de l'intestin irritable (colopathie), Reflux Gastro-Œsophagien, Reflux Gastro-Œsophagien, Autres dont chirurgie.

5- Maladies dysimmunitaires et hématopoïétiques : Aplasie médullaire et autres cytopénies chroniques, déficit immunitaire primitif grave nécessitant un traitement prolongé, Infection par le virus de l'immuno-déficience humaine, Hémoglobinopathies, hémolyses, chroniques constitutionnelles et acquises sévères, Hémophilies et affections constitutionnelles de l'hémostase graves, Affection maligne du tissu lymphatique.

6- Maladies nephro et genito-urinaires : Népropathie chronique grave et syndrome néphrotique primitif, Maladie sexuellement transmissible récente, Infections urinaires récidivantes (plus de 4 épisodes par an), Autres.

7- Maladies neurologiques : Formes graves des affections neurologiques et musculaires (dont myopathies), épilepsie grave, Maladie d'Alzheimer et autres démences, Maladie de Parkinson, Sclérose en plaques, Autres (Ex: Sclérose latérale amyotrophique, …).

8- Maladies systémiques et auto-immunes : Maladie de Horton, Pseudopolyarthrite rhizomélique, Périarthrite noueuse, Lupus érythémateux aigu systémique, Sclérodermie systémique, Polyarthrite rhumatoïde, Spondylarthrite ankylosante, Rhumatisme psoriasique, Autre.

9- Maladies ostéo-articulaires : Ostéoporose, Arthrose, Goutte, Chondrocalcinose articulaire, Autre.

10- Affections psychiatriques de longue durée : Oui, Non. Si oui : champs libre

11- Cancer (Tumeur maligne) : Sein, Poumon, Prostate, Colon et rectum, Estomac, Pancréas, Vésicule et voies biliaires, Foie (primitif ou secondaire), Utérus, ORL, Vessie, Peau (Si peu : mélanome ou autre), Os, Autres.

12- Transplantation d'organe : Oui, Non. Si oui : en clair

13- Allergies: Allergie alimentaire (Ex: arachides, fruits de mer, etc.), Allergie aérienne (Ex: pollen, acariens, moisissures, etc.), Allergie cutanée (Ex: latex, produits chimiques, médicaments, produits cosmétiques, etc.), Allergie médicamenteuse (Ex : antibiotiques, anti-inflammatoires, etc), Autres

14- Autre maladie chronique diagnostiquée (Ex: Psoriasis, maladie de Verneuil, …)

17. Prenez-vous des médicaments régulièrement (tous les jours, toutes les semaines ou à une autre fréquence) ?

* Oui / Non. Si oui, nom des médicaments à indiquer

18. Avez-vous subi une appendicectomie (intervention chirurgicale consistant à retirer l'appendice, généralement en raison d'une appendicite) ?

* Oui – Non -- Je ne sais pas

19. Au cours des 7 derniers jours, Indiquez la fréquence des symptômes digestifs suivants :

* Douleurs abdominales ou inconfort abdominal

* Ballonnements intestinaux

* Flatulence (émission de gaz)

* Borborygmes / gargouillements de l’estomac

* Jamais, 1 fois / semaine, 2-3 fois/semaine, 4-6 fois/semaine, tous les jours

20. Quelle est votre fréquence habituelle de selles par semaine ?

* Plus de 2 par jour, Une ou 2 tous les jours, Environ 4-6 par semaine, Environ 2-3 par semaine, Environ 1 par semaine

21. Souffrez-vous régulièrement de constipation ?

* Oui / Non

22. Souffrez-vous régulièrement de diarrhée ?

* Oui / Non

23. Quelle est l'apparence habituelle de vos selles ?

* Score Bristol 1-7 (échelle)

24. Avez-vous eu des infections à répétition (plus de 2 fois) au cours de la dernière année (ORL, urinaire, pulmonaire ou gynécologique)?

* Oui / Non.

* Si oui Menus déroulant (choix multiples): ORL (rhinopharyngites aiguës, otites moyennes aiguës, angines, sinusites, autres), Urinaire, Pulmonaire, Gynécologique

25. Au cours du dernier mois, avez-vous senti que les choses allaient comme vous le vouliez ?

* Jamais, Presque jamais, Parfois, Assez Souvent, Très souvent

26. Au cours du dernier mois, combien de fois avez-vous trouvé que les difficultés s’accumulaient à un tel point que vous ne pouviez les surmonter ?

* Jamais, Presque jamais, Parfois, Assez Souvent, Très souvent

27. Au cours du dernier mois, vous a-t-il semblé difficile de contrôler les choses importantes de votre vie ?

* Jamais, Presque jamais, Parfois, Assez Souvent, Très souvent

28. Au cours du dernier mois, vous êtes-vous sentis confiants dans vos capacités à prendre en main vos problèmes personnels ?

* Jamais, Presque jamais, Parfois, Assez Souvent, Très souvent

29. A quel point êtes-vous satisfait(e) de votre sommeil ?

Très satisfait – Satisfait – Moyennement satisfait – Insatisfait – Très insatisfait

Vos habitudes alimentaires :

30. Excluez-vous des aliments de votre alimentation ?

Les produits contenant du gluten -- Les produits laitiers -- Les fruits à coque -- Les œufs -- Le poisson -- Le soja -- Les crustacés (crevettes, crabes, homards, etc.) -- Les produits contenants de l’arachide -- Autre (-> champs libre)

31. Quel est votre régime alimentaire ?

Omnivore (je mange de tout) -- Ovo-lacto-vegetarien (je ne mange pas de viande mais je consomme des oeufs et du lait) -- Pesco-ovo-lacto-végétarien (je ne mange pas de viande mais je consomme du poisson, des fruits de mer, des oeufs et du lait) -- Flexitarien (je mange occasionnellement de la viande ou du poisson) -- Pescétarien (je mange du poisson et des crustacés comme seules sources de protéines animales) -- Ovo-pescétarien (je mange des œufs, du poisson et des crustacés comme seules sources de protéines animales) -- Végétalien / Vegan (je ne mange aucun produit d'origine animale, ni viande, ni lait, ni œufs, …) -- Je ne sais pas -- Autre (-> champ libre)

32. PAR JOUR, combien de portions de légumes mangez-vous ?

Portions à montrer

33. AU COURS DE L'ANNEE PASSEE, quels sont les légumes que vous avez eu l'habitude de manger ?

Liste de légumes à cocher

34. PAR JOUR, combien de portions de fruits mangez-vous ?

Portions à montrer

35. AU COURS DE L'ANNEE PASSEE, quels sont les fruits que vous avez eu l'habitude de manger ?

Liste de fruits à cocher

36. AU COURS D'UNE SEMAINE, quels sont les légumineuses (légumes secs) que vous avez l'habitude de manger ?

Liste de légumineuses à cocher

37. PAR SEMAINE, combien de portions de légumineuses / graines / noix mangez-vous ?

Portions à montrer

38. PAR JOUR, combien de portions de féculents non complets mangez-vous ?

Portions à montrer

39. PAR JOUR, combien de portions de féculents complets mangez-vous ?

Portions à montrer

40. AU COURS D'UNE SEMAINE, combien de portions de viande ou poisson ou d'œufs mangez-vous ?

41. Et plus particulièrement pour le POISSON, AU COURS D'UNE SEMAINE, combien de portions mangez-vous ?

Portions à montrer

42. AU COURS D'UNE SEMAINE, consommez-vous les produits laitiers ou substituts ci-dessous ?

Liste à cocher

43. PAR JOUR, plus particulièrement pour les produits laitiers ALLEGES ou à faible teneur en matières grasses, combien de portions mangez-vous ?

Portions à montrer

44. En moyenne, combien de plats cuisinés/ transformés (plats à réchauffer au micro-onde, pizzas, cordon bleu, pâté en croute…) consommez-vous?

3 par jour -- 2 par jour -- 1 par jour -- 4-6 par semaine -- 2-3 par semaine -- moins d’un par semaine -- 0

45. PAR JOUR, combien de portions de sucreries et sucres ajoutés mangez-vous ?

Portions à montrer

46. AU COURS D'UNE SEMAINE, quels sont les produits sucrés que vous avez l'habitude de manger ?

Liste à cocher

47. PAR JOUR, combien de portions de matières grasses et d'huiles ajoutées mangez-vous ?

Portions à montrer

48. AU COURS D'UNE SEMAINE, quelles huiles utilisez-vous le plus souvent pour la cuisson ?

Liste à cocher

49. AU COURS D'UNE SEMAINE, quelles huiles utilisez-vous le plus souvent pour l'assaisonnement ?

Liste à cocher

50. AU COURS D'UNE SEMAINE, quelles boissons consommez-vous quotidiennement ?

Liste à cocher

51. PAR JOUR, combien de verres d’eau consommez-vous ?

Portions à montrer

52. AU COURS D'UNE SEMAINE, combien de verres d’alcool en moyenne buvez-vous ?

Portions à montrer

53. Avez-vous l’habitude de consommer des compléments alimentaires?

Oui / Non.

Si oui :

- Menu déroulant : Vitamines, Minéraux, Extraits de plantes, Probiotique (Ex : Bifidobacterium, Lactobacillus, levure), Prébiotique (Ex : inuline), Autres
- En consommez-vous actuellement ? Oui / Non
