# Supplementary material for: Associations Among Diet, Health, Lifestyle, and Gut Microbiota Composition in the General French Population: Protocol for the Le French Gut – Le Microbiote Français Study
Source: JMIR Res Protoc. 2025 May 13;14:e64894. doi: 10.2196/64894 (PMC12117270; doi:10.2196/64894)
Supplement: Multimedia Appendix 4 [file resprot_v14i1e64894_app4.docx]

Appendix 2 - Executive and steering committee

The executive committee, led by INRAE, is composed of INRAE staff and AP-HP staff, including the Coordinating Investigator. It meets at least once a month. The executive committee ensures the operational and financial management of the project under the authority of the steering committee. The steering committee is composed of one member per partner. It meets twice a year. The steering committee is the decision-making and arbitration body of the projects.
